# Management of abortion complications at a rural hospital in Uganda: a quality assessment by a partially completed criterion-based audit

**DOI:** 10.1186/s12905-015-0233-y

**Published:** 2015-09-20

**Authors:** Natja Mellerup, Bjarke L. Sørensen, Gideon K. Kuriigamba, Martin Rudnicki

**Affiliations:** Department of Obstetrics and Gynecology, Odense University Hospital, Odense, Denmark; Centre for Innovative Medical Technology, University of Southern Denmark, Odense, Denmark; Department of Surgery, Makerere University college of Health sciences, Kampala, Uganda

## Abstract

**Background:**

Complications of unsafe abortion are a major contributor to maternal deaths in developing countries. This study aimed to evaluate the clinical assessment for life-threatening complications and the following management in women admitted with complications from abortions at a rural hospital in Uganda.

**Methods:**

A partially completed criterion-based audit was conducted comparing actual to optimal care. The audit criteria cover initial clinical assessment of vital signs and management of common severe complications such as sepsis and haemorrhage. Sepsis shall be managed by immediate evacuation of the uterus and antibiotics in relation to and after surgical management. Shock by aggressive rehydration followed by evacuation.

In total 238 women admitted between January 2007 and April 2012 were included. Complications were categorized as incomplete, threatened, inevitable, missed or septic abortion and by trimester. Actual management was compared to the audit criteria and presented by descriptive statistics.

**Results:**

Fifty six per cent of the women were in second trimester. Abortion complications were distributed as follows: 53 % incomplete abortions, 28 % threatened abortions, 12 % inevitable abortions, 4 % missed abortions and 3 % septic abortions. Only one of 238 cases met all criteria of optimal clinical assessment and management. Thus, vital signs were measured in 3 %, antibiotic criteria was met in 59 % of the cases, intravenous fluid resuscitation was administered to 35 % of women with hypotension and pain was managed in 87 % of the cases. Sharp curettage was used in 69 % of those surgically evacuated and manual vacuum aspiration in 14 %. In total 3 % of the abortions were categorized as unsafe. Two of eight women with septic abortion had evacuation performed during admission-day, one woman died due to septic abortion and one from severe haemorrhage.

**Conclusions:**

Guidelines were not followed and suboptimal assessment or management was observed in all but one case. This was especially due to missing documentation of vital signs necessary to diagnose life-threatening complications, poor fluid resuscitation at signs of shock, and delayed evacuation of septic abortion.

## Background

Complications of abortion is the second highest cause of maternal death worldwide and may cause 13 % of all pregnancy-related deaths [1, 2]. Globally, 21 of 200 million annual pregnancies are terminated by unsafe abortion [1]. This figure has remained unchanged for the last decades [3]. The World Health Organization (WHO) defines unsafe abortion as ‘a procedure for terminating an unintended pregnancy either by individuals without the necessary skills or in an environment that does not conform to minimum medical standards, or both’ [4]. This definition may change according to a recent WHO bulletin as home administration of misoprostol is increasingly accessible as a safe abortion method [5].

Abortion rates are higher in countries with restrictive abortion laws [6, 7] and in Sub-Saharan Africa 97 % of the abortions are estimated to be unsafe [2, 3]. In South Africa the abortion law was liberalized in 1994, which was followed by a decrease in the incidence of abortion related infections by 52 % and the mortality by over 90 % [8]. In Uganda, as in the majority of Sub-Saharan African countries, induced abortion is illegal unless the pregnancy is a risk to the woman’s life. Still, the most recent estimate indicates that 54 induced abortions per 1000 women of reproductive age were performed in 2003, and 28 % of these women were treated for complications [9]. Up to 50 % of Ugandan women will hereby receive treatment for complications of an induced abortion during their life [9]. Septic abortion and haemorrhage are the most frequent life-threatening complications of unsafe abortion, and in 2008 it was estimated that 26 % of maternal deaths in Uganda were accounted as abortion related complications [10].

In order to improve the circumstances of unsafe abortion, a ‘post abortion care’ (PAC) model has been developed and implemented in several countries with restrictive abortion laws, including Uganda [11]. Post abortion care focuses on better access to family planning methods and emergency care.

Manual vacuum aspiration (MVA) has been introduced in Uganda as a part of PAC. It has proved to be a safe method to evacuate first trimester abortions. When MVA is unavailable blunt curettage is recommended for first trimester abortions and always for second trimester abortions [12]. Sharp curettage (SC) is discouraged as it increases the risk of uterine perforations [12, 13]. Septic abortion is mainly caused by unsafe aseptic technique and retained intrauterine products. The infection can rapidly become life threatening and must be treated immediately at time of diagnosis by evacuation of the uterus [12]. MVA has improved access to emergency obstetric care as mid-level health providers can perform it safely even in peripheral health facilities [14].

A second method to improve access to PAC and the level of care is the use of the prostaglandin E1 analogue misoprostol for medical abortions [12, 15]. The method should be recommended for both first and second trimester abortions. Incomplete abortion and missed abortion with no signs of sepsis or severe haemorrhage can be managed safely with misoprostol and surgical procedures should be avoided [12]. The higher availability of misoprostol has also resulted in usage of the drug for illegal abortions [2, 5], though it is thought that this may decrease the frequency of complications [2].

Even though PAC has been introduced [11] and WHO regularly publishes guidelines for management of abortion complications in developing countries [4, 12, 16] it is essential that the actual management performed is optimal and meets the recommendations [12, 17].

Criterion-based audit (CBA) has been used successfully in high-income countries to improve care in order to avoid complications during pregnancy, research suggests that the same applies in low- and middle-income countries [18, 19].

The objective of this study was by a retrospective partially completed criterion-based audit to evaluate the clinical assessment for life-threatening complications and the following management in women admitted with complications from abortion at a rural hospital in Uganda.

## Methods

### Settings

This study was carried out at a rural hospital in South-western Uganda. The hospital serves a population of 100,000 inhabitants providing 40,000 consultations annually. Clinical officers were responsible for the outpatient department where the women initially were assessed and management initiated. Two medical officers were in charge of the maternity ward, the adult ward, the HIV-department, the outpatient department and the surgical ward. Only medical officers performed the uterine evacuations. The maternity ward was further staffed with midwives, nurse-midwives and nurses. Nursing staff was present 24 hours a day and a medical officer was on call during night shifts. Induced abortion “on demand” was not performed at the hospital. Contraceptives were offered for free at family planning counselling before discharge.

### Population

The sample size included all women admitted during a five-year retrospective period from January 2007 to April 2012. This period was chosen as the resources and opportunities to manage abortion complications at the hospital did not change during this period. Thus, uterine evacuation could not be performed prior to the inclusion period. Furthermore, the five-year period may eliminate selection bias such as seasonal variation in pregnancy incidence, opportunity to reach the hospital and variations in staff composition and experience.

### Inclusion and exclusion criteria

**Fig. 1:**
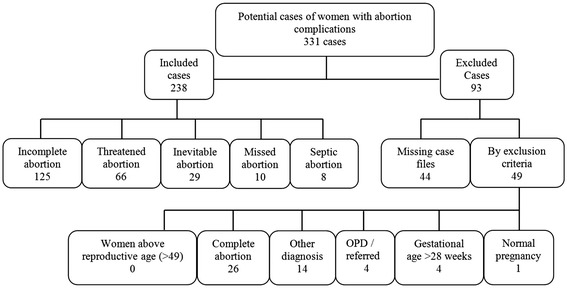
Research material collected by inclusion of five abortion types and defined exclusion criteria. OPD: outpatient department

Inclusion criteria were all women registered as admitted with incomplete, threatened, inevitable, missed and septic abortion. Cases were excluded if management only took place at the outpatient department, the women were referred during treatment or the abortion showed to be complete (Fig. 1). In these cases the results would be misleading since full management could not be assessed. Women with a gestational age above 28 weeks were excluded as deliveries from this point are considered a birth in Uganda. The diagnosis and gestational age was based on ultrasound in less than one third of the cases although not routinely used for threatened abortion. In cases where ultrasound was not applied the gestation age was established by last normal menstrual period or uterine size estimated by clinical or medical officers.

### Criterion-based audit cycle

Steps one to three of the classic five-step CBA cycle were performed. These steps consist of establishment of criteria for good quality care, data collection and analysis of the findings. We could not complete the audit cycle by implementation of changes (step four) and re-evaluation (step five) because of time constraints. Preliminary results were instead presented to the staff and recommendations on how to improve practice in the future were discussed in plenary.

### Step one - establishment of criteria for good quality care

Step one was to establish realistic and relevant criteria for optimal assessment and management of abortion complications. The hospital’s local standards for management turned out to be inaccurate, missing, incomplete and remarkably different from international standards (Table 1). Thus, manual vacuum aspiration was only part of the hospital’s guideline regarding “unsafe abortion”, and the guideline did not mention the importance of immediate evacuation at signs of sepsis or excessive bleeding, but only that it should be considered after stabilization of the patient with IV fluids and antibiotics. Furthermore, oxytocin and ergometrine should not be used for medical abortion and finally fluid resuscitation at a blood pressure < 100 mmHg should be managed with two litres of fast running fluids.

**Table 1:** The hospital’s guideline for management of abortion

Initial assessment	Asses for shock and sepsis (fever, foul smelling discharge, hypotension, tachycardia). Give NS, at least 500 ml.
Incomplete abortion	<16 weeks and slight to moderate bleeding: use fingers or ring forceps to remove products protruding through the cervix.
	<16 weeks and bleeding is heavy: evacuation by SC. If evacuation is not immediately possible: 0.2 mg ergometrine IM or 400 mcg misoprostol orally.
	>16 weeks: 20 IU oxytocin in 1 L IV NS until expulsion of POC.If necessary, give 200 mcg misoprostol vaginally every 4 hour until expulsion, max 800 mcg. Evacuate any remaining POC^a^
Threatened abortion	Admit and treat with antibiotics. If abdominal pain, give analgesia.
Inevitable abortion	<16 weeks: give 10 IU oxytocin and plan for evacuation^a^.
	>16 weeks, no active bleeding^b^: await spontaneously expulsion. Control pain. If necessary, infuse oxytocin 20 IU in 1 L IV NS.
Unsafe abortion	Assess for complications, injuries and sepsis. If blood pressure > 100 mmHg give 500 ml NS, if < 100 mmHg 1 L. Give antibiotics. When stable, consider MVA.

A guideline for missed abortion did not exist

*IM* intramuscular, *IU* international units, *IV* intravenous, *L* litre, *mcg* micrograms, *mg* milligrams, *ml* millilitres, *mmHg* millimetres of mercury, *MVA* manual vacuum aspiration, *NS* normal saline, *POC* products of conception, *SC* sharp curettage

^a^No recommendation for type of evacuation procedure

^b^No recommendations for procedure for inevitable abortion above 16 weeks of gestational age and active bleeding

Our criteria were selected based on acceptability, simplicity, feasibility and critical importance after reviewing international guidelines and national guidelines (Table 2) [12, 16, 17]. The national standards are published by the Ugandan Ministry of Health and based on WHO’s guidelines [17]. The medical doctor in charge of the hospital ensured prior to the data collection that our audit criteria were realistic according to the local setting.

**Table 2:** Audit Criteria for acceptable management

1. Assessment of vital signs^a^:
Measure blood pressure, pulse, temperature^b^ and respiration frequency.
2. Fluid resuscitation:
At a systolic blood pressure ≤ 90 mmHg: infuse two litres of fast running normal saline.
3. Broad-spectrum antibiotics:
Give antibiotics at: temperature ≤ 36° or ≥ 38° Celsius, purulent foul smelling vaginal discharge, surgical evacuation or unsafe abortion.
4. Evacuation of retained intrauterine products:
Medical evacuation: misoprostol. Surgical evacuation: manual vacuum aspiration or blunt curettage.
For gestational age ≤ 12 weeks surgical evacuation shall be performed with manual vacuum aspiration.
Expectant management is only recommended when the well-informed patient wish to use this method and no additional complications are present.
5. Pain management:
Any expression of pain shall result in analgesics to the patient.
6. Waiting time for evacuation at sepsis or heavy bleeding:
Acute emergency evacuation of the uterus must be performed. This shall not be delayed by expectant antibiotic treatment but performed immediately.

^a^To be measured at admission. Oxygen saturation is an important vital to monitor, but the ward did not have the equipment to perform the measurement

^b^Axil measurement, 0.5° was therefore added to the raw data to compensate

### Step two – data collection

The data collection was performed from February to April 2012. Potential eligible cases were identified by provisional diagnosis from the maternity ward’s admission-register, e.g. abortion, vaginal bleeding or abdominal pain. Based on the admission date from the register, we could identify the medical records anonymously from the archive and include or exclude due to the final diagnosis. To identify cases either not registered in the admission-books or registered differently in the admission book and the medical file all gynaecological case files in the archive were cross-checked. The contraceptive care registration system showed major differences compared to the maternity ward’s register, and consequently we could not reliably identify the women’s post-abortion contraceptive use. Accordingly, this resulted in the decision to exclude family planning as a part of the partially completed CBA. A standardized data collection form was pre-made and used to screen the medical records. In case a procedure was not documented, it was assessed as ‘not performed’. The staff did not ask the women consistently whether they had had an illegal induction of the abortion, but when the women informed the staff it was documented in the medical file.

### Step three - analysis of the findings

The third step was to compare the actual practice to the selected audit criteria. After the data collecting period the hospital received the data collection form and was encouraged to continue the cycle by implementing the suggested changes and re-evaluate the management.

### Statistics

Data were continuously entered into Microsoft Access. IBM SPSS 20.0 was used to calculate mean, median and standard deviation**.**

### Ethics

The hospital and its personnel have been kept anonymous. Ethical permission from the hospital to conduct the study was obtained prior to the study from the hospital´s authorities and made available for review by the Editor of this journal. The Danish National Committee on Biomedical Research Ethics in Denmark have assessed the project and found that no further ethical approvals for the analyses were necessary according to Danish law.

## Results

We identified 331 potential cases. Of these, 238 met the inclusion criteria (Fig. 1). Registered abortion complications were distributed as follows; incomplete abortion 52.5 %, threatened abortion 27.7 %, inevitable abortion 12.2 %, missed abortion 4.2 %, and septic abortion 3.4 %.

Missing cases accounted for 44/331 (13.3 %). Twenty-one (47.7 %) of these had septic abortion as provisional diagnosis compared to 8 (3.4 %) in the included cases.

Population characteristics are presented in Table 3. More than one in five had previously had an abortion and 55.5 % of the women were in second trimester mainly according to last menstrual period. Ultra-sound was used to determine gestational age in 70 women (29.4 %).

**Table 3:** Population characteristics

Women’s characteristics	
Age, mean ± SD	26 ± 6.9
Marital status^a^, n (%)	
Married	140 (59.3)
Single	2 (0.8)
nd	94 (39.8)
Trimester^b^, n (%)	
1	70 (33.2)
2	117 (55.5)
nd	24 (11.4)
HIV infection, n (%)	
Yes	7 (3.0)
No	106 (44.9)
Deny test	4 (1.7)
nd	119 (50.4)
Previous abortion, n (%)	
Yes	54 (22.9)
No	78 (33.1)
nd	104 (44.1)
Gravida^c^, median ± SD	3 ± 3
Parity^c^, median ± SD	1 ± 3
Gravida and (parity), n	
0	0 (57)
1	47 (39)
2	42 (22)
3	20 (15)
4	19 (10)
5+	56 (40)

*nd* not documented, *SD* standard deviation

^a^Two cases of incomplete abortion were readmissions due to insufficient prior management. The demographic data, except trimester, are only included for the first admission

^b^Two cases admitted with inevitable and 25 threatened abortion are not included in ‘trimester’; the pregnancy continued

^c^Not documented for all cases

One case (1/238) strictly fulfilled all the audit criteria (Table 4).

**Table 4:** Assessment for life-threatening complications and management

Fulfilment of the criteria	Incomplete		Threatened		Inevitable		Missed		Septic		Total	
	*n* = 125		*n* = 66		*n* = 29		*n* = 10		*n* = 8		*n* = 238	
	n	%	n	%	n	%	n	%	n	%	n	%
Vital signs												
Yes	4	3.2	2	3.0	1	3.4	0	0.0	0	0.0	7	2.9
No	121	96.8	64	97.0	28	96.6	10	100.0	8	100.0	231	97.1
Fluid resuscitation												
Yes	11	47.8	0	0.0	1	25.0	0	0.0	1	50.0	13	35.1
No	12	52.2	8	100.0	3	75.0	0	0.0	1	50.0	24	64.9
Antibiotics												
Yes	86	68.8	30	45.5	11	37.9	5	50.0	8	100.0	140	58.8
No	39	31.2	36	54.5	18	62.1	5	50.0	0	0.0	98	41.2
Evacuation												
Yes	28	25.7	2	13.3	2	13.3	4	50.0	0	0.0	36	23.5
No	81	74.3	13	86.7	13	86.7	4	50.0	6	100.0	117	76.5
Analgesia												
Yes	71	78.0	55	96.5	20	90.9	7	100.0	7	100.0	160	87.
No	20	22.0	2	3.5	2	9.1	0	0.0	0	0.0	24	13.0
All criteria												
Yes	1	0.8	0	0.0	0	0.0	0	0.0	0	0.0	1	0.4
No	124	9.2	66	100.0	29	100.0	10	100.0	10	100.0	237	99.6

### Assessment of vital signs

Temperature was recorded for 194 (81.5 %), blood pressure for 163 (68.8 %), pulse for 96 (40.3 %) and respiration frequency for eight (3.4 %) women. All four vital signs were measured in seven women (2.9 %) (Table 4).

### Fluid resuscitation

A systolic blood pressure ≤ 90 mmHg or less was recorded in 37 (15.5 %) women while intravenous fluid was not administered to 24 (64.9 %) of these. The criteria for fluid resuscitation were met by 35.1 % (Table 4). Seven (87.5 %) of the eight diagnosed with sepsis did not receive intravenous fluids.

### Administration of broad-spectrum antibiotics

Antibiotic treatment was indicated in 140 cases and received by 134 (95.7 %), while 92 out of 98 (93.9 %) without indication had prescribed antibiotics. In total 58.8 % thereby met the criteria for antibiotics (Table 4).

### Evacuation of retained intra-uterine products

Surgical evacuation was performed in 98 women. Fourteen (14.3 %) were evacuated by MVA, and of these five (35.7 %) were in second trimester. Sixty-eight (69.4 %) were evacuated by SC, and of these 37 (54.4 %) were in second trimester. Three women (3.0 %) were evacuated by blunt curettage. The surgical evacuation method was not documented in 13 cases (13.3 %). Thirty (62.5 %) of the surgically evacuated second trimester abortions were above 15 week of gestation. Medical evacuation was used in 80 women and of these misoprostol was used in 41 cases (51.3 %). The remaining cases (48.7 %) received oxytocin, ergometrine or combinations of these with or without misoprostol. One septic abortion was tried evacuated by oxytocin. In total 36 cases (23.5 %) met the criteria for evacuation of retained intrauterine products (Table 4).

### Analgesia

The criteria for pain management was met in 160 women (87 %) (Table 4).

### Timing for evacuation at sepsis or heavy bleeding

Timing for evacuation at signs of sepsis or excessive bleeding could not be evaluated due to lack of documentation. Bleeding amount was poorly documented and time was only documented by date. Six women (75 %) with septic abortion had surgical evacuation of retained products. Two were evacuated during admission day, two were postponed one day, and two women were evacuated two days after their admission.

Ten women (4.2 %) had an unsafe abortion. One had consulted a private clinic, two had unknown herbal medicine installed in the birth canal, one had wild grass in the cervix, one had triggered the abortion with unknown medicine and one had received an unknown injection. No information was available regarding the method for the last four women. One woman had a traumatized cervix but denied to have induced the abortion. The case fatality rate was 2/238 (0.84 %). One woman died due to an unsafe septic abortion. In this case SC was postponed until two days after admission. Another woman died due to haemorrhage although she received fluid resuscitation and blood transfusions, but she had no evacuation of the retained products.

## Discussion

This is to our knowledge the only partially completed CBA study evaluating the quality of clinical assessment for life-threatening complications and management of abortion related complications in a low-income country.

Our study identified suboptimal care in both the initial clinical assessment for life-threatening complications and the following management in all but one case, not only according to our criteria, but even the hospital’s own guidelines were not followed. This lack of consensus may cause misdiagnosis and incorrect treatment based on the clinicians’ preference. Life-threatening complications may be overlooked, as well as failure to perform fluid resuscitation and evacuation of the uterus at signs of shock and sepsis. These deficiencies are related to maternal death [20, 21]. Furthermore, the method used in more than half of the evacuations are discouraged due to an increased risk of uterine perforation [12, 13].

Recently it was documented that women who died from abortion complications waited significantly longer for treatment than patients with eclampsia and post-partum haemorrhage [22]. Likewise, others have shown that the achieved awareness of this delay in treatment led to a major reduction in treatment delay as well increased willingness to improve practice [23]. These observations are in agreement with ours demonstrating that postponement of treatment may result in adverse outcome. Delayed management was observed in both cases of maternal death in our study and these may have been avoided if the midwives performed immediate MVA, which they can perform as safely as medical doctors [14]. Unfortunately, MVA was not available due to lack of sterilizing equipment, which is a well-known reason for substandard care [13, 21, 24, 25]. However, the mortality rate we observed was comparable to other studies in similar settings [26, 27].

Furthermore, our results documented that the personnel were either unaware of guidelines or were not capable of following them. The results also documented an inadequate regular update of the local guidelines. Thus, a major informative work is necessary to improve knowledge and the use of guidelines, especially if this is a common problem in rural areas and it contributes to the maternal mortality from abortions.

The strength of the study is the systematic approach of the parts of CBA with criteria established beforehand minimizing the risk of bias from subjective assessment. A Cochrane review including 19 studies evaluating CBA in low-income countries reported improved obstetric management in 18 studies, though not in relation to post abortion care [28]. However, our study has some limitations. We did not complete the audit cycle and consequently the possible impact on improving care cannot be observed. Further, it relies fully on the written documentation available. The reliability of the documented data should be questioned, because what was actually done might not have been written down. Furthermore, our study could not systematically document if complications were due to unsafe abortions, as this was not systematically documented.

Thus, our observations represent only the audited hospital. It is impossible to say to what extent our observations are comparable to the management of abortion complications in developing countries in general. Completing the CBA and assessing several hospitals would have strengthened our results.

The hospital in our study used premade sheets at admittance where only temperature had to be documented and consequently, we observed this parameter to be the most frequently documented vital sign. Further, we observed a high proportion of missing cases and nearly half of them were recorded as septic abortion at admission. This observation questions a report from Pirkle et al. [29] who indicates that missing files were mostly random and was anticipated not to affect the results. This discrepancy from the study of Pirkle et al. [29] may be due to the poor documentation and consequently incorrect provisional diagnosis.

In two hospital-based studies from Tanzania 52 % to 63 %, respectively of women with abortion complications admitted to have had an unsafe abortion, when interviewed with an empathetic approach [30, 31]. This large number of women undergoing unsafe abortion contrasts our study. We observed few unsafe abortions and this can explain the surprisingly low number of septic abortions. One explanation might be the more widespread use of misoprostol, which has been described in several studies [2, 5]. Although this may limit the number of unsafe abortions and consequently sepsis, it is impossible to assess whether an informal use of misoprostol exists in this geographic area. Thus this phenomenon needs to be explored more intensively. Another explanation to the low number of septic abortions could be the poor documentation and that some septic patients may have been overlooked due to missing recording of vital data.

It is interesting that more than half of the cases in our study were in second trimester whereas most spontaneous abortions takes place in the first trimester. It must be kept in mind however, that gestational age can be inaccurate when established by fundal height or last normal menstrual period as compared to ultra-sound as gold standard [32].

We suggest that future audits on complications of abortions include prospective data collection, timing of events and contraceptive counselling. There is an urgent need to upgrade the admittance sheet with fields for documentation of the necessary parameters to diagnose life-threatening conditions. Subsequently, a clear flowchart for management should be developed to improve practice and consistency of treatment and finally a structured case file system may act to avoid the missing cases.

The staff’s awareness of the actual substandard management [23] combined with delegating responsibilities to the personnel, e.g. the development of the flowchart and re-evaluation of the implemented changes, may increase the motivation to raise the standards for clinical practice.

Furthermore, we suggest that future audits complete the audit cycle in order to observe potential improvement in actual management and patient outcomes. Such a study needs to be locally anchored and the success will depend on the motivation of staff and time consumption [19]. In order to increase the success rate we suggest that an audit only includes a few central criteria like vital signs, fluid resuscitation at low blood pressure, emergency evacuation at signs of septic abortion or excessive bleeding and contraceptive counselling.

## Conclusion

Our study revealed that guidelines were not followed resulting in suboptimal care. This was especially due to missing documentation of vital signs necessary to diagnose life-threatening complications, poor fluid resuscitation at signs of shock, and delayed evacuation of the uterus at septic abortion. In order to optimize suboptimal care we suggest that future audits are completed and carried out prospectively with local ownership. Improved motivation may be achieved by delegation of the responsibility locally thereby increasing the usefulness of the admittance sheet. Creation of a flowchart may increase implementation of any changes thereby ensuring better clinical practice.

## Abbreviations

CBA: Criterion-based audit
MVA: Manual vacuum aspiration
SC: Sharp curettage
WHO: World Health Organization
